# Characterization of two *PEBP* genes, *SrFT* and *SrMFT*, in thermogenic skunk cabbage (*Symplocarpus renifolius*)

**DOI:** 10.1038/srep29440

**Published:** 2016-07-08

**Authors:** Yasuko Ito-Inaba, Hiromi Masuko-Suzuki, Haruhiko Maekawa, Masao Watanabe, Takehito Inaba

**Affiliations:** 1 Organization for Promotion of Tenure Track, University of Miyazaki, 1-1 Gakuenkibanadai-nishi, Miyazaki 889-2192, Japan; 2 Graduate School of Life Sciences, Tohoku University, 2-1-1 Katahira, Aoba-ku, Sendai 980-8577, Japan; 3 Department of Agricultural and Environmental Sciences, Faculty of Agriculture, University of Miyazaki, 1-1 Gakuenkibanadai-nishi, Miyazaki 889-2192, Japan

## Abstract

Floral thermogenesis has been found in dozens of primitive seed plants and the reproductive organs in these plants produce heat during anthesis. Thus, characterization of the molecular mechanisms underlying flowering is required to fully understand the role of thermogenesis, but this aspect of thermogenic plant development is largely unknown. In this study, extensive database searches and cloning experiments suggest that thermogenic skunk cabbage (*Symplocarpus renifolius*), which is a member of the family Araceae, possesses two genes encoding phosphatidyl ethanolamine-binding proteins (PEBP), *FLOWERING LOCUS T (SrFT*) and *MOTHER OF FT AND TFL1 (SrMFT*). Functional analyses of *SrFT* and *SrMFT* in Arabidopsis indicate that *SrFT* promotes flowering, whereas *SrMFT* does not. In *S. renifolius*, the stage- and tissue-specific expression of *SrFT* was more evident than that of *SrMFT. SrFT* was highly expressed in flowers and leaves and was mainly localized in fibrovascular tissues. In addition, microarray analysis revealed that, within floral tissues, *SrFT* was co-regulated with the genes associated with cellular respiration and mitochondrial function, including *ALTERNATIVE OXIDASE* gene proposed to play a major role in floral thermogenesis. Taken together, these data suggest that, among the *PEBP* genes, *SrFT* plays a role in flowering and floral development in the thermogenic skunk cabbage.

Floral thermogenesis has been found in dozens of flowering plants including basal angiosperms (Nymphaeaceae and Schisandraceae)^1^,^2^,^3^,^4^, magnoliids (Magnoliaceae)^5^,^6^,^7^, monocots (Araceae)^8^,^9^,^10^, and eudicots (Nelumbonaceae and Rafflesiaceae)^10^,^11^. According to the updated Angiosperm Phylogeny Group Classification (APG III)^12^, basal angiosperms are the most primitive group of angiosperms, and the magnoliids diverged early. In addition, the Araceae and Nelumbonaceae are early diverging monocots and eudicots, respectively. Heat production is not restricted to angiosperms but also occurs in several families of gymnosperms (i.e., Cycadaceae and Zamiaceae)^10^,^13^,^14^, which are more primitive than angiosperms among seed plants. Thus, it is reasonable to suggest that plant species that are able to produce heat in their reproductive organs belong to relatively primitive plant families. These plants differ from well-studied annual herbaceous plants, in aspects of growth and development, in that they flower after several years of vegetative growth and exhibit thermogenesis when they bloom. Thus, it is of interest to determine the mechanisms by which thermogenic plants initiate flowering, progress through floral development, and begin thermogenesis.

Extensive studies on the roles of floral thermogenesis in physiology, ecology and pollination biology have suggested that thermogenesis may be related to reproductive functions such as attracting pollinators by spreading odor^15^, providing a warm environment for pollinators^16^, and protecting the inflorescences from freezing damage^17^. In addition, extensive studies using arum species have revealed that thermogenesis is positively correlated with oxygen consumption rate^10^,^18^. However, the physiological roles of floral thermogenesis and the causal relationship between thermogenesis and respiration have not been completely established due to the lack of molecular genetic studies on thermogenic arum species and techniques to control their flowering.

Thermogenic skunk cabbage (*Symplocarpus renifolius*), which is a member of the family Araceae, is a thermoregulatory plant that can maintain spadix temperatures at approximately 20 °C for 1–2 weeks in early spring, when the ambient temperature falls below freezing^19^. *S. renifolius* is a polycarpic plant that flowers after several years of vegetative growth (Fig. 1a,b). The inflorescences begin heat production when they bloom (Fig. 1d), and terminate it when the pollen is released from the anthers (Fig. 1e). Interestingly, floral development precedes leaf development in the adult phase (Fig. 1c–f). In *S. renifolius*, the developmental features of the flowers and leaves generally contrast those of well-studied plants, in which flowering and floral development occur after leaf development. During the floral development from the thermogenic stage (Fig. 1d) to the post-thermogenic stage (Fig. 1e), intracellular structures within the floral tissues change significantly; the mitochondrial content is reduced, especially in the petals and pistils, whereas the vacuolar volume increased^20^,^21^. Consistent with the cellular change, the genes involved in cellular respiration and mitochondrial function are up-regulated in the former thermogenic spadices, whereas the genes involved in stress responses and protein degradation are up-regulated in the latter post-thermogenic spadices^21^,^22^. To uncover the molecular mechanisms underlying floral thermogenesis, much effort has been made to characterize an alternative oxidase (AOX), an energy-dissipating mitochondrial protein, and AOX has been proposed to play a pivotal role due to the correlation between heat production and AOX concentration as well as activity^23^,^24^,^25^,^26^,^27^.

Plant phosphatidyl ethanolamine-binding proteins (PEBP) family is divided into three main clades, *FLOWERING LOCUS T (FT*)-like, *TERMINAL FLOWER1 (TFL1*)-like, and *MOTHER OF FT AND TFL1 (MFT*) -like^28^. These members have been extensively studied in Arabidopsis, especially FT and TFL1. Despite an amino acid similarity of over 88%, these two proteins have antagonistic functions; FT promotes flowering by mediating both photoperiod and temperature signals, whereas TFL1 represses flowering^29^,^30^,^31^. The remaining *MFT*-like clade is generally thought to be the evolutionary ancestor to the other two clades^32^,^33^. A first duplication event resulted in two families of plant *PEBP* genes (*MFT*-like and *FT/TFL1*-like), and the second duplication resulted in the *FT*-like and *TFL1*-like clades. Thus, the *MFT*-like clade contains not only angiosperms but also more primitive plants, including mosses, spikemosses and gymnosperms, whereas the *FT*/*TFL1*-like clade contains only angiosperms. Given that the evolution of plant *PEBP* genes seems to coincide with the evolution of seed plants, it is of interest to determine which *PEBP* genes exist in skunk cabbage since it is presumably one of the earliest diverging monocots among angiosperms.

Recent molecular studies have shown that, in several species, FT and its orthologs that are translated in the leaves act as florigens^34^,^35^,^36^. In Arabidopsis, FT moves to the shoot apical meristem (SAM) via the phloem and, once there, forms a transcriptional complex with a basic leucine zipper (bZIP) transcription factor, FD. Transcription of floral regulator genes, such as *APETALA1 (AP1*), is then activated, leading to flowering^37^. Thus, as the major function, *FT*-like genes have been proposed to control the switch from the vegetative phase to the reproductive phase. However, recent functional studies have demonstrated more divergent roles for *FT*-like genes. In sugar beet, for example, two orthologous *FT* genes, *BvFT1* and *BvFT2*, exhibit antagonistic functions; *BvFT2* is essential for flowering, whereas *BvFT1* represses flowering, in both transgenic sugar beet and Arabidopsis plants^38^. In potato, two orthologous *FT* genes, *SP3D* and *SP6A*, have different functions, as well; *SP3D* promotes flowering, but *SP6A* promotes tuberization^39^. Furthermore, in Arabidopsis, *AtFT* is expressed in guard cells and regulates stomatal opening^40^. Therefore, *FT* seems to be involved in various other processes, in addition to flowering.

In this study, we characterized the expression and function of two *PEBP* genes, *FT* and *MFT*, designated as *SrFT* and *SrMFT*, from thermogenic skunk cabbage. Our study suggests that *SrFT* may play a principal role in the regulation of flowering in thermogenic skunk cabbage, and the expression of *SrFT* mRNA in floral tissues may have additional roles in processes other than flowering, through the complicated gene network associated with energy metabolism.

## Results

### Isolation and identification of *SrFT* and *SrMFT* cDNA from *S. renifolius*

An *S. renifolius* cDNA fragment library was searched to identify the DNA sequences of orthologous *PEBP* genes, including *FT*-like, *TFL1*-like, and *MFT*-like. Two *PEBP* genes, *FT* and *MFT*, were found in the cDNA fragment library, but *TFL1* homologs and other *FT/MFT* isoforms could not be found. To isolate *SrFT* and *SrMFT* genes from the tissues of *S. renifolius*, cloning primers were designed based on the *SrFT* and *SrMFT* sequences, and RT-PCR was performed. The amplified 600-bp region of *SrFT* cDNA contained a 525-bp protein coding sequence (LC030437) flanked by a 21-bp 5′-untranslated region and a 54-bp 3′-untranslated region. The predicted SrFT protein consisted of 174 amino acid residues and had a calculated molecular mass of 19.6 kDa. In contrast, the amplified 700-bp region of *SrMFT* cDNA contained a 639-bp protein coding sequence (LC030438) flanked by a 33-bp 5′-untranslated region and a 28-bp 3′-untranslated region. The predicted SrMFT protein consisted of 212 amino acid residues with a calculated molecular mass of 22.9 kDa. To isolate *TFL1-like* genes from *S. renifolius*, RT-PCR was performed using the degenerate primers (Supplementary Figs 1a,c, and 2); however, no fragments were amplified. The amplification of other *PEBP* genes using the other sets of degenerate primers were also unsuccessful (Supplementary Fig. 1b).

The SrFT showed significant homologies to *Cymbidium* and *Oncidium* FT proteins (87% and 85%, respectively; Fig. 2a). SrFT was also highly homologous to rice and Arabidopsis FT proteins (83% and 76%, respectively). In addition, the conserved key amino acid residues Tyr and Gln in FT homologs, which contribute to FT function, were identified at positions 84 and 139 of the SrFT protein, respectively (Fig. 2a). In Arabidopsis, mutational and structural analyses of chimeric proteins revealed that two amino acid residues are possibly the most critical for distinguishing FT and TFL1 activity^29^,^30^,^31^. However, the key amino acids were not conserved in SrMFT, leading to the hypothesis that SrMFT does not play a central role in the regulation of flowering. The SrMFT sequence exhibited 57% homologies to the *Glycine max* MFT protein (GmMFT) and contained an amino acid extension at its N-terminus that was similar to GmMFT (Fig. 2a). The sequence was also orthologous to rice and Arabidopsis MFT proteins (54% and 55%, respectively).

A phylogenetic tree for the *PEBP* genes was constructed using several amino acid sequences of other FT/TFL orthologs (Fig. 2b), and other MFT orthologs (Fig. 2c). The tree was divided into four major clades: FT, TFL, BFT (BROTHER OF FT AND TFL1), and MFT clades. The SrFT protein was located in the monocot group of the FT clade and was also located close to the FT proteins from orchids. Although *FT* genes have been isolated from numerous plant species, *SrFT* is the first to be isolated from a primitive flowering plant (i.e., *S. renifolius*). According to the updated APG III^12^, the Araceae, which includes *S. renifolius*, seems to be more ancient than other plant families that are included in the monocot group of FT sequence. The SrMFT protein was also located in the monocot group of the MFT clade but was also located close to the MFT proteins from grasses.

### Expression analyses of *SrFT* and *SrMFT* mRNA in *S. renifolius*

To investigate the distribution of *SrFT* mRNA, semi-quantitative RT-PCR was performed using various tissues of *S. renifolius* at the thermogenic female stage. *SrFT* mRNA was highly expressed in flowers and leaves but not in roots or spathes (Fig. 3a). To further examine the expression profile of *SrFT* mRNA in the flower, semi-quantitative RT-PCR was also performed using different floral tissues, including petals, pistils, stamens and the pith of female-stage flowers (Fig. 3b). *SrFT* was expressed in all floral tissues, but the expression was slightly lower in stamens and pith than in petals and pistils.

Next, the stage-specific expression of *SrFT* mRNA in flowers was examined using the spadices of different developmental stages in *S. renifolius* (Fig. 3c,d). During the former stage of floral maturation, levels of *SrFT* mRNA increased and reached a peak in mature flowers (Fig. 3c), and then, during the late stages of floral maturation, SrFT levels remained high (Fig. 3d). We also examined the seasonal changes in *SrFT* mRNA in leaves, using semi-quantitative RT-PCR (Fig. 3e). We found that *SrFT* mRNA was expressed in all leaves examined here, and was also more highly expressed during the reproductive stage in winter and spring than in early summer.

In contrast to *SrFT, SrMFT* transcripts were constitutively expressed in various tissues and developmental stages, except that the level of *SrMFT* was higher in female-stage spadices than in the other stages investigated. We also investigated the expression of the *TFL2*-like gene, designated as *SrTFL2*. SrTFL2 contained highly conserved regions among Arabidopsis and apple TFL2 proteins (Supplementary Fig. 3). Although TFL2 does not belong to the PEBP family, the protein is active in the regulation of flowering in both Arabidopsis^41^,^42^ and apple^43^. *SrTFL2* transcripts were also constitutively expressed in various tissues and developmental stages, but interestingly, during the former stage of floral maturation, *SrTFL2* transcripts decreased contrary to the increase of *SrFT* mRNA levels (Fig. 3c). Therefore, instead of *TFL1*-like genes, *SrTFL2* may function in *S. renifolius*.

### *In situ* localization of *SrFT* mRNA in *S. renifolius*

In Arabidopsis and rice, *FT* mRNA is localized in cotyledons but not in meristems^35^,^44^, but there have been very few studies of the *in situ* localization of *FT* mRNA in mature floral organs. Thus, we carried out *in situ* hybridization using an antisense probe to examine the tissue-specific localization of *SrFT* mRNA in detail. In floral tissues (Fig. 4a–d), the signals derived from *SrFT* mRNA were mainly observed in the vascular bundles in petals (Fig. 4a), pistils (Fig. 4b), stamens (Fig. 4c), and the outer layer of the pith (Fig. 4d). In leaf tissues (Fig. 4e,f), *SrFT* mRNA was also mainly localized in vascular bundles. A sense probe used as the control did not produce any signals in flowers (Fig. 4g) and leaves (Fig. 4h). Together, these results indicated that *SrFT* mRNA was localized mainly in the vascular tissues of flowers and leaves.

### Microarray analysis of *SrFT* and other co-regulated genes

To examine the expression profiles of *SrFT* and co-regulated genes in mature floral tissues, microarray analysis was carried out using labeled cRNA that had been prepared from different floral tissues (Fig. 5). Consistent with RT-PCR analyses (Fig. 3b), the expression of *SrFT* mRNA was found to be higher in petals and pistils than in stamens and pith. Interestingly, we observed that the tissue-specific expression of *SrFT* was quite similar to that of *SrAOX* (Fig. 5a). The microarray result of *SrAOX* mRNA was validated with RT-PCR (Supplementary Fig. 7b at the bottom). Thus, to identify additional co-regulated genes with these two genes, we performed cluster analysis using a Poisson approach. As a result, 6523, 7259, 7867, and 5535 genes were grouped into clusters I, II, III, and IV (Supplementary Fig. 4). Two genes, *SrFT* and *SrAOX*, were classified into cluster IV along with the other co-regulated genes (Supplementary Table 4).

To further assess the major function of these co-regulated genes in the cellular processes associated with thermogenesis in *S. renifolius*, 8279 genes that could be annotated with the Arabidopsis Gene Identifier (AGI) codes were analyzed using Gene Ontology analysis (Fig. 5b,c). Interestingly, the percentage of genes encoding mitochondrial proteins in cluster IV was the highest among the four clusters, and the percentage of genes associated with electron transport or the energy pathway in cluster IV was also the highest among the four clusters (Fig. 5c and Supplementary Tables 5 and 6). Among the four clusters, transcripts that encoded chloroplast and plastid proteins as well as transporters were also overrepresented in cluster IV (Fig. 5c). These data suggest that the expression of *SrFT* is co-regulated with other genes associated with energy metabolism.

### Analysis of Arabidopsis transformants expressing *SrFT* or *SrMFT*

*FT* genes isolated from many plants exhibit an *FT*-like function in Arabidopsis; however, *Picea abies*, which is a primitive seed plant, has *PEBP* genes that occupy an intermediate phylogenetic position between the *FT*-like and *TFL1*-like (FT/TFL1-like) genes and exhibit a *TFL1*-like function^32^. Thus, it is of interest to determine whether the *FT* genes of skunk cabbage, an ancestral flowering plant, encode an activity that promotes flowering. To examine *SrFT* function, *SrFT* and *SrFT-GFP* cDNA constructs driven by the CaMV 35S promoter were individually transformed into Arabidopsis plants. In the transgenic lines, the *35S::SrFT* (#4-1 and #10-3) and *35S::SrFT-GFP* (#12-3) constructs caused very early flowering and the *35S::SrFT-GFP* (#14-1) constructs caused modestly early flowering, compared with the empty vector (Fig. 6a).

To assess the flowering response quantitatively, the number of days required for the main shoot to attain a length of 1 cm (days to bolting) and the number of rosette leaves observed on that day were measured for ten plants from each transgenic line (Fig. 6b[Fig f6]c, and Table 1). In agreement with Fig. 6a, three transgenic lines (*35S::SrFT* #4-1, and #10-3, and *SrFT-GFP* #12-3) exhibited a very early flowering phenotype, and another line (*35S::SrFT-GFP* #14-1) exhibited a modestly early flowering phenotype, compared to that of the wild type.

To explore the correlation of the early flowering phenotype with *SrFT* expression in the *35S::SrFT* and *SrFT-GFP* transgenic lines, RT-PCR was performed. As shown in Fig. 6d, the expression of *SrFT* mRNA was observed in the transgenic lines, but not in wild type. To analyze the expression of SrFT protein, SrFT-GFP proteins expressed in rosette leaves of two *SrFT-GFP* transgenic lines (#12-3 and #14-1) were detected using immunoblotting with anti-GFP antibodies (Fig. 6e). The levels of SrFT-GFP protein were higher in #12-3 than #14-1. This result suggests that the very early flowering phenotype observed in *35S::SrFT-GFP* (#12-3) is due to higher SrFT protein expression.

We also measured the days to bolting and the numbers of rosette leaves observed on that day using *35S::SrMFT* and *35S::SrMFT-GFP* lines (Fig. 7 and Table 1). The results showed that SrMFT did not promote flowering in Arabidopsis.

Taken together, these data clearly show that SrFT proteins promote flowering and that elevated levels of SrFT protein cause earlier flowering in Arabidopsis.

## Discussion

In this study, we isolated two *PEBP* genes, *SrFT* and *SrMFT*, from thermogenic skunk cabbage (*S. renifolius*). Although skunk cabbage is a primitive seed plant, the primary structure of the proteins encoded by the two genes was similar to those found in other plants. *SrFT* mRNA was highly expressed in both flowers and leaves, and its level in flowers significantly increased during floral maturation. In contrast, *SrMFT* mRNA was constitutively expressed in various tissues and stages. Interestingly, within floral tissues, *SrFT* and several genes related to mitochondrial function and cellular respiration, including *SrAOX*, had similar expression profiles. The expression of *SrFT* in Arabidopsis resulted in an early flowering phenotype, indicating that *SrFT* promotes flowering, whereas the expression of *SrMFT* in Arabidopsis failed to promote flowering. Taken together, these data suggest that, among *PEBP* genes, *SrFT* plays a role in the regulation of flowering and floral development in the thermogenic skunk cabbage, through the complicated gene network associated with energy metabolism.

**Figure 1:**
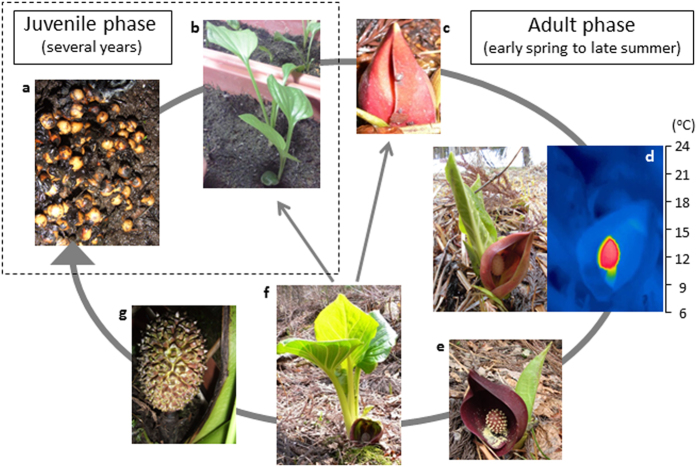
The life cycle of skunk cabbage (*Symplocarpus renifolius*). *S. renifolius* is a polycarpic plant that flowers after several years of vegetative growth. In early spring, the flowers begin producing heat when they bloom and terminate heat production when the pollen is released from the anthers. At the end of spring, leaf development is initiated at floral senescence, and environmental conditions determine whether or not flowers will bloom in the following year. In summer, if pollination is successful, the flower becomes a fruit, which contains many seeds. (**a**) Seeds, (**b**) seedlings, (**c**) immature-stage flowers (pre-thermogenic stage), (**d**) female-stage flowers (thermogenic stage), (**e**) male-stage flowers (post-thermogenic stage), (**f**) developed leaves, and (**g**) fruits. The thermal image of the right panel in (**d**) was taken using an FLIR SC 620 thermal imager (FLIR SYSTEMS).

**Figure 2:**
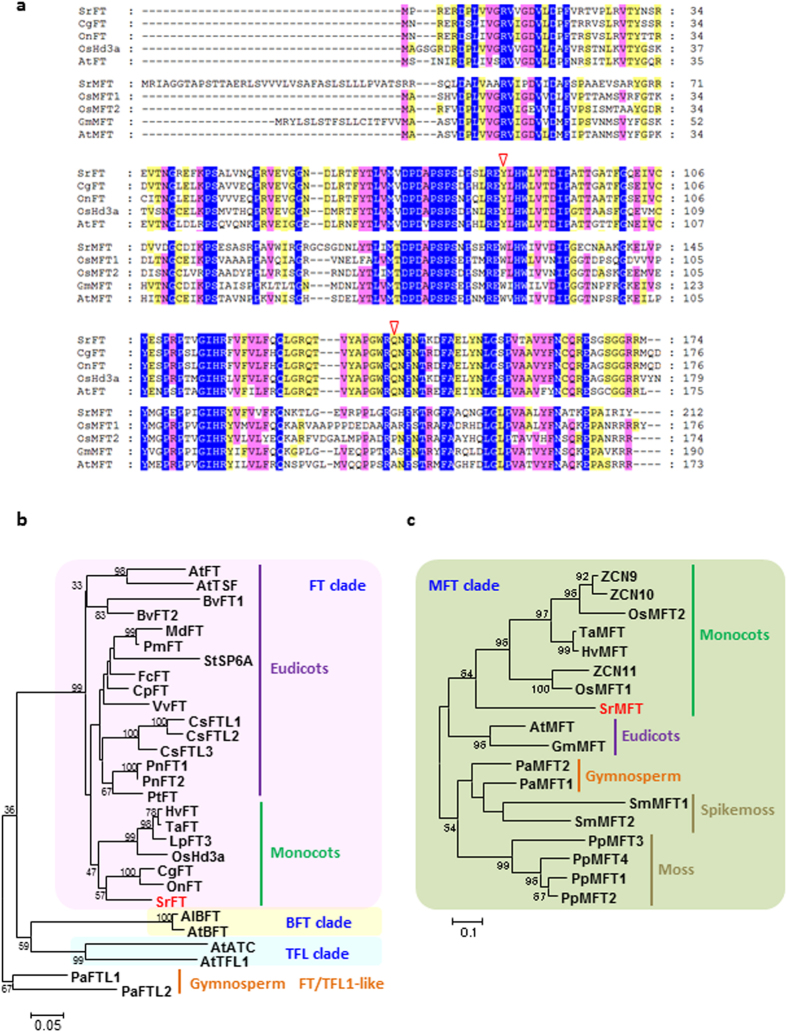
Sequence analysis of SrFT, SrMFT, and related proteins. (**a**) Alignment of the predicted amino acid sequences of SrFT, SrMFT, and related proteins. The predicted SrFT sequence was aligned with CgFT (*Cymbidium goeringii*), OnFT (*Oncidium Gower* Ramsey), OsHd3a (rice), and AtFT (Arabidopsis). The SrMFT sequence was aligned with OsMFT1 and 2 (rice), GmMFT (*Glycine max*), and AtMFT (Arabidopsis) sequences. Amino acids highlighted in blue, pink, or yellow are conserved across all, more than eighty percent, or more than half of the protein sequences in the alignment, respectively. Arrowheads indicate amino acids (Y84 and Q139) that are critical to FT function. (**b**) A phylogenetic tree of the predicted amino acid sequences of SrFT and related proteins. (**c**) A phylogenetic tree of the predicted amino acid sequences of SrMFT and related proteins. The tree was created using MEGA 6.06 with the Clustal W and maximum likelihood method. Bootstrap values above 30 are shown near each branch. The consensus phylogenic trees are shown with bootstrap values from 1000 replications. The accession numbers, locus IDs, and species abbreviations are listed in Supplementary Table 2.

**Figure 3:**
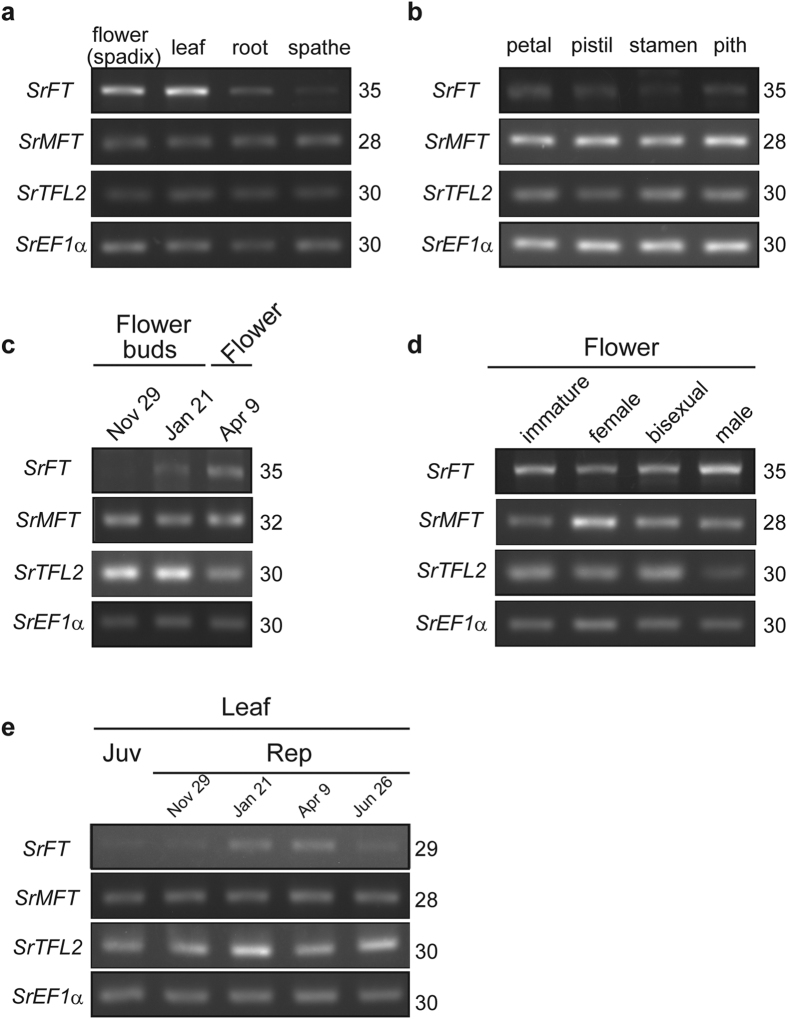
The tissue- or stage-specific expression of two *PEBP* genes, *SrFT* and *SrMFT*. (**a**) Tissue-specific expression in plant tissues. (**b**) Tissue-specific expression in floral tissues. (**c**) Stage-specific expression in flowers during the transition from floral buds to mature flowers. (**d**) Stage-specific expression in flowers at the late stage of floral maturation, including immature (pre-thermogenesis), female (strong thermogenesis), bisexual (weak thermogenesis), and male (post-thermogenesis) stages. (**e**) Stage-specific expression in leaves of juvenile (Juv)- and reproductive (Rep)-phase plants. To prevent saturation, semi-quantitative RT-PCR was analysed with different PCR cycles (Supplementary Fig. 7) and the appropriate numbers of PCR cycles were shown on the right side of each gel. The signals detected here were not from contaminated genomic DNA (Supplementary Fig. 8). In (**c,e**), potted plants were used to collect samples (Supplementary Fig. 9).

**Figure 4:**
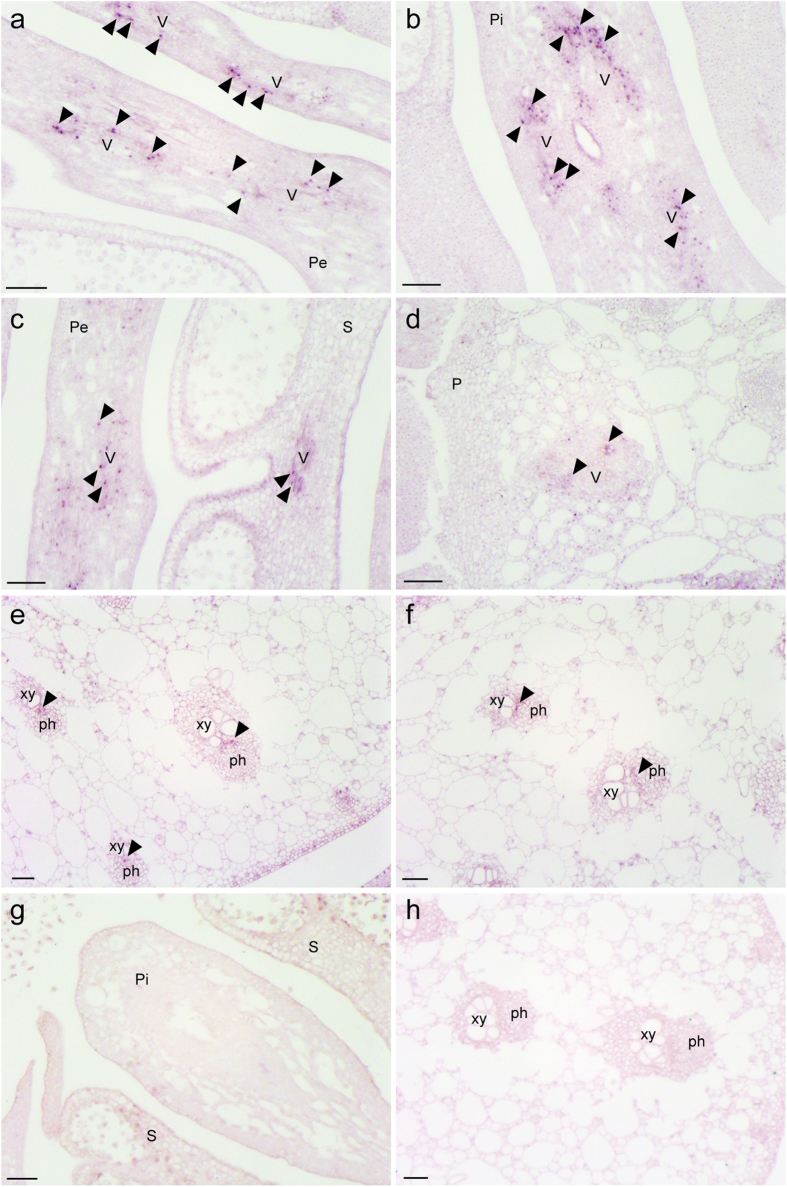
Localization of *SrFT* mRNA in flowers and leaves. DIG-labeled antisense RNA probes were hybridized on cross-sections of flowers (**a–d**) and leaves (**e,f**). As the control, sense RNA probes were hybridized on cross-sections of flowers (**g**) and leaves (**h**). Arrowheads indicate the locations of signals. Pe, petal; Pi, pistil; P, pith; S, stamen; V, vascular bundle; xy, xylem; ph, phloem. Bar =100 μm.

*S. renifolius* is a polycarpic plant that flowers after several years of vegetative growth (Fig. 1). Once the plant flowers, it can bloom almost every year under optimal environmental conditions, and one of the most interesting features of *S. renifolius* is that floral development precedes leaf development in the adult phase (Fig. 1c–f). In *S. renifolius*, the flower blooms in early spring, whereas leaf development begins at the end of spring. The developmental features in the flowers and leaves contrast those of well-studied plants, in which flowering and floral development occurs after leaf development. In the present study, the expression of *SrFT* was high in both flowers and leaves; therefore, we hypothesize that *SrFT* expressed in each organ has distinct functions. In leaves, the SrFT protein is likely to function as a florigen, and we speculate that SrFT is transported to the SAM, where it stimulates the formation of flower buds that will mature in the following season. However, in flowers, *SrFT* may regulate floral or fruit maturation. This proposal is consistent with the observation that the level of *SrFT* mRNA increased during the maturation of floral buds (Fig. 3c). Highly expressed *FT* genes in floral tissues were observed not only in skunk cabbage (Figs 3 and 4) but also in Arabidopsis (Supplementary Fig. 6), and the levels of *FT* mRNA increased significantly from flower buds to mature flowers in both plants. Thus, highly expressed *FT* genes in these tissues may have additional roles in floral development in various plants. In addition, although it would be of interest to study the levels and the localization of *SrFT* mRNA during the transition from the vegetative stage to the reproductive stage, we did not undertake such a study because of the difficulty in culturing plants in laboratory conditions during this transition.

The plant *PEBP* gene family is divided into three main clades: *FT*-like, *TFL1*-like, and *MFT*-like. Among *PEBP* genes, we identified *SrFT* and *SrMFT* as the major skunk cabbage genes of the *FT*-like and *MFT*-like subfamilies, respectively. However, there were no fragments of *TFL1*-like genes in our skunk cabbage cDNA database, and we could not amplify any *TFL1-like* fragments using RT-PCR and degenerate primers (Supplementary Fig. 1). Interestingly, gymnosperms possess only two types of *PEBP* genes, *MFT*-like and a group that occupies an intermediate phylogenetic position between the *FT*-like and *TFL1*-like (*FT/TFL1*-like)^32^. It is, therefore, of great interest to determine whether there are two or three types of *PEBP* genes in skunk cabbage. However, the complete sequence of the skunk cabbage genome will be needed to determine whether *TFL1*-like genes exist or not. Interestingly, in the skunk cabbage inflorescences, the levels of *SrFT* mRNA increased during floral maturation, whereas the levels of *SrTFL2* mRNA decreased (Fig. 3c). Since *TFL2*, which does not belong to the PEBP family, functions in the repression of flowering in Arabidopsis^41^,^42^ and apple^43^, *TFL2*, instead of *TFL1*, may regulate flowering and floral development together with *SrFT* in thermogenic skunk cabbage.

In Arabidopsis and rice, *FT*/*Hd3a* expression is much higher in leaves than in the shoot apex, and is observed in phloem tissues of both cotyledons and leaves^35^,^44^,^45^. Based on the expression studies and analyses of transgenic Arabidopsis/rice expressing GFP-tagged FT/Hd3a, it became clear that FT/Hd3a proteins are produced in the leaves and subsequently move to the shoot apex, where they promote flowering^34^,^35^. Interestingly, the number of studies describing *FT* transcripts in tissues other than leaves has increased recently. For example, in grapes, *VvFT* is highly expressed in inflorescences and developing fruit and is especially prominent in developing seeds^46^, and in poplar trees, *PnFT1* and *PnFT2* are highly expressed in capsules^47^. In addition, the expression levels of *OnFT* and *CgFT* in orchids are significantly higher in young flower buds and decrease during floral maturation^48^,^49^. Although *OnFT* and *CgFT* exhibited the highest homologies to *SrFT*, their expression patterns were in marked contrast to those of skunk cabbage. *SrFT* expression in skunk cabbage increased during floral maturation (Fig. 3c), whereas *FT* expression in orchids decreased. In addition, two potato *FT* genes, *SP3D* and *SP6A*, also exhibit different functions; *SP3D* promotes flowering, whereas *SP6A* promotes tuberisation^39^, and accumulates to high levels in stolons and leaves of short-day induced plants. These observations suggest that FT functions may be more diverse than previously thought and that other functions may be found with future investigation.

**Figure 5:**
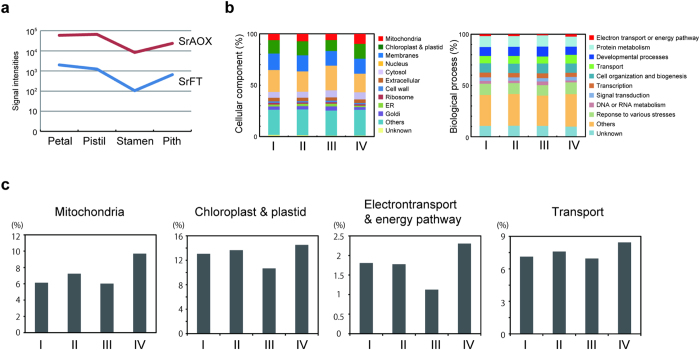
Microarray analysis of four different floral tissues. (**a**) *SrFT* and *SrAOX*, representative genes for flowering and respiration/thermogenesis, respectively, had similar expression profiles among the different floral tissues. (**b**) 27,184 genes were classified into four clusters (I-IV) according to their expression profiles in the different floral tissues. Of the resulting 6523, 7259, 7867, and 5535 genes in clusters I, II, III and IV, respectively, 2052, 1981, 2438, and 1808 were annotated with AGI codes, respectively. The annotated genes were classified based on the GO annotation that assigns cellular components and biological processes to each sequence. (**c**) Categories that had the highest percentage representation in cluster IV of all clusters were extracted. Most notably, many genes involved in mitochondrial function and in electron transport and the energy pathway were found in cluster IV.

**Figure 6 f6:**
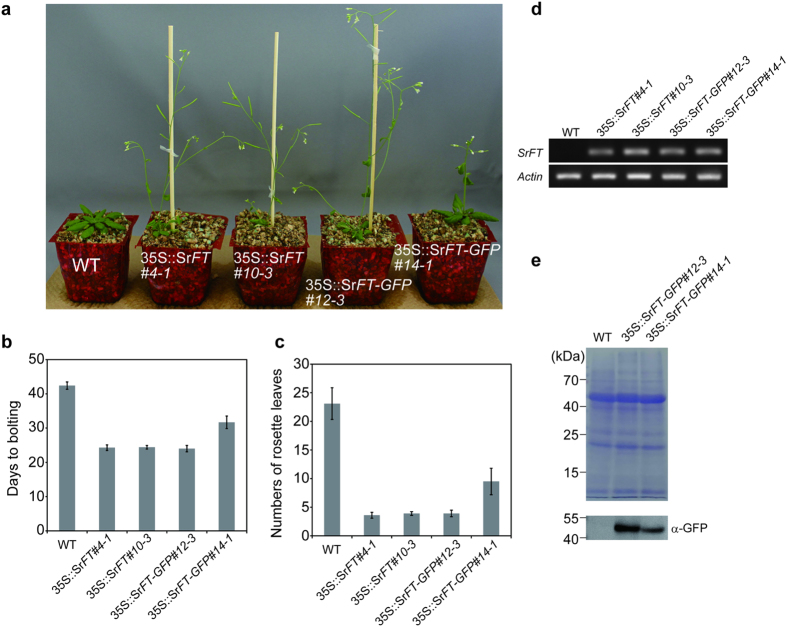
Analysis of transgenic Arabidopsis plants that ectopically expressed *SrFT* or *SrFT-GFP*. (**a**) Phenotypes of 33-day-old wild type (empty vector), *35S::SrFT*, and 3*5S::SrFT-GFP* transgenic plants grown in soil under LD conditions. (**b,c**) Flowering response of wild type, *35S::SrFT*, and *35S::SrFT-GFP* transgenic plants in soil under LD conditions. Data are means ± standard deviation (SD; N = 10). (**d**) *SrFT* mRNA expressed in rosette leaves of 33-day-old wild type, *35S::SrFT*, and *35S::SrFT-GFP* transgenic plants were analyzed using RT-PCR. Actin is shown as a loading control. (**e**) Rosette leaves of 33-day-old wild type and *35S::SrFT-GFP* transgenic plants were analyzed using SDS-PAGE and then visualized using CBB-staining or immunoblotting with α-GFP antibodies.

**Figure 7:**
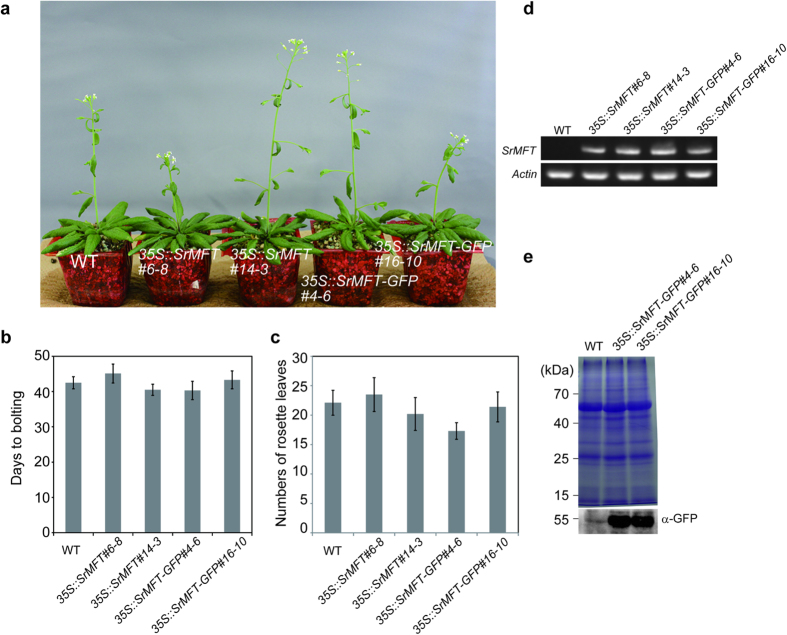
Analysis of transgenic Arabidopsis plants that ectopically expressed *SrMFT* or *SrMFT-GFP*. (**a**) Phenotypes of 46-day-old wild type (empty vector), *35S::SrMFT*, and 3*5S::SrMFT-GFP* transgenic plants grown in soil under LD conditions. (**b,c**) Flowering response of wild type, *35S::SrMFT*, and *35S::SrMFT-GFP* transgenic plants in soil under LD conditions. Data are means ± standard deviation (SD; N = 10). (**d**) *SrMFT* mRNA expressed in rosette leaves of 29-day-old wild type, *35S::SrMFT*, and *35S::SrMFT-GFP* transgenic plants were analyzed using RT-PCR. Actin is shown as a loading control. (**e**) Rosette leaves of 29-day-old wild type and *35S::SrMFT-GFP* transgenic plants were analyzed using SDS-PAGE and then visualized using CBB-staining or immunoblotting with α-GFP antibodies.

Extensive research on the expression and activity of AOX has shown that the protein plays a pivotal role in floral thermogenesis^23^,^24^,^25^,^26^,^27^. In contrast, several studies have suggested that uncoupling protein (UCP), which plays a major role in mammalian thermogenesis, likely plays a minor role in plant thermogenesis^23^,^25^. Interestingly, we found that *SrAOX* transcripts were co-regulated with *SrFT* transcripts within floral tissues (Fig. 5a). Classification analysis based on gene expression profiles also revealed that *SrAOX* and *SrFT* were classified into cluster IV (Supplementary Table 4). In addition, *SrMFT* was also co-regulated with *SrAOX* and *SrFT* and classified into cluster IV; however, *SrUCPA* was not co-regulated with these genes and was classified into cluster II (Supplementary Table 4 and Supplementary Fig. 5). In previous studies, genes involved in cellular respiration and mitochondrial function were significantly enhanced during the female stage of skunk cabbage floral development^21^,^22^. Of these genes, several were classified into cluster IV in this study, and were co-regulated with *SrFT* and *SrAOX* (Supplementary Fig. 5). Thus, defining the mechanisms that underlie the co-regulation of the genes involved in flowering and respiration in floral tissues will be a future research objective.

Photoperiodism in plants from the Araceae family, including skunk cabbage, remains largely unknown. Only a few plants, such as *Spirodera polyrhiza* (duckweed), are known to be day-neutral plants with respect to turion formation^50^. However, since skunk cabbage has a long juvenile phase and is buried under heavy snow during winter, it is difficult to examine the effects of environmental factors, including day length, on the timing of flowering. It is also difficult to control the flowering of skunk cabbage by changing environmental conditions. Thus, altering endogenous conditions by overexpressing *SrFT* is probably the best strategy to promote skunk cabbage flowering. To achieve this goal, recent advances in transformation techniques and tissue culture will be useful; especially since the number of reports using these techniques in the Araceae family has been gradually increasing^51^,^52^,^53^. In addition, since floral thermogenesis is always observed in reproductive organs, such as flowers and inflorescences, technical advances in the control of flowering will elucidate the molecular mechanisms that underlie floral thermogenesis and will likely reveal many unanswered questions.

## Methods

### Plant materials

Skunk cabbages, *Symplocarpus renifolius*, tissues were sampled from plants growing in the marshlands of Iwate and Nagano, Japan with the exception of a few potted plants that were transferred from outdoors at the end of autumn, in order to study gene expression in floral buds.

### cDNA sequencing

A cDNA fragment library was prepared from *S. renifolius* by using a GS FLX Titanium Rapid Library Preparation Kit (Roche Diagnostics), and over 450,000 cDNA fragments were sequenced with an FLX+ genome sequencer (Roche Diagnostics). Sequences that were highly homologous to the *FT* and *MFT* of several plant species were screened from ~35,000 contigs using BLAST-X searches, and cloning primers were designed to isolate the genes. *TFL1* homologs and other *PEBP* genes were not identified in these datasets. However, partial sequences of *TFL2* homologs were identified (Supplementary Fig. 3), and primers were designed to analyze their gene expression.

### Cloning of *FT* and *MFT* genes from *S. renifolius*

Total RNA was extracted from leaves of *S. renifolius* using the RNeasy Plant Mini Kit (QIAGEN), and cDNA was synthesized from 1 μg total RNA using a PrimeScript^TM^ RT reagent kit (Takara Bio Inc.). The resulting cDNA was then used as the template in subsequent RT-PCR experiments with KOD-Plus DNA polymerase (TOYOBO). For the cloning of *SrFT* genes, nested-PCR was carried out using two sets of primers: SrFT-F4 and SrFT-R4 for 1^st^ PCR, and SrFT-F1 and SrFT-R1 for 2^nd^ PCR. To clone the *SrMFT* genes, 5′-RACE was conducted using a FirstChoice RLM-RACE Kit (Life Technologies) and the gene-specific outer and inner primers SrMFT-R3 and SrMFT-R4, respectively, which was designed from partial *SrMFT* sequences on our cDNA fragment library. The full-length clone of *SrMFT* was isolated using RT-PCR and the primers SrMFT-F5 and SrMFT-R1. After being cloned into the pZErO-2 vector, the full-length *SrFT* and *SrMFT* genes were sequenced using the primers M13F and M13R. The nucleotide sequences of *SrFT* and *SrMFT* have been submitted to DNA Data Bank of Japan (DDBJ) with accession number LC030437 and LC030438, respectively. Although considerable effort was made to isolate *TFL1* homologs and other *PEBP* genes (Supplementary Figs 1 and 2), these genes were not isolated. All primers used in this study are summarized in Supplementary Table 1.

### Construction of plant transformation vectors

To generate the 35S::*SrFT* construct, the PCR products that were amplified using the primers SrFT-F1 and SrFT-R1 were cloned into the *Nco*I-*Nhe*I site of the pCAMBIA 1301 vector (pCAM1301) under the control of the cauliflower mosaic virus (CaMV) 35S promoter using an In-Fusion HD Cloning Kit w/Cloning Enhancer (Clontech Laboratories, Inc). The pCAM1301 vector was partially digested because it contains two *Nhe*I digestion sites. To generate the 35S::*SrFT-GFP* construct in a similar way, PCR was carried out using the primers SrFT-F1 and SrFT-R2. The *GFP* gene was amplified from pUC35S::GFP using PCR with KOD-Plus DNA polymerase (TOYOBO) and the primers GFP-F1 and GFP-R1. The two amplified PCR products were mixed and cloned into the *Nco*I-*Nhe*I site of pCAM1301 using an In-Fusion HD Cloning Kit w/Cloning Enhancer (Clontech Laboratories, Inc), and the resulting DNA inserts were sequenced with the primers pCAM1301-F1 and pCAM1301-R1. To generate *35S::SrMFT* and *35S::SrMFT-GFP* constructs, the pZerO-2 vector that contained the *SrMFT* gene was used as a template. The *SrMFT* gene for *35S::SrMFT* was amplified using the primers SrMFT-IF-F1 and SrMFT-IF-R1, and the *SrMFT* gene for *35S::SrMFT-GFP* was amplified using the primers SrMFT-IF-F1 and SrMFT-IF-R2.

### Arabidopsis transformation

All pCAMBIA constructs were introduced into Arabidopsis (ecotype Columbia) via *Agrobacterium tumefaciens*-mediated transformation, using the floral dip method^54^. The flowering time of the T3 line was evaluated.

### Molecular phylogenetic and evolutionary analyses

The predicted amino acid sequences of FT/MFT and other related proteins from angiosperms were collected by searching the National Center for Biotechnology Information (NCBI) databases. In addition, the protein sequences of MFT orthologous genes from gymnosperms (*P. abies*), spikemosses (*S. moellendorffii*), and mosses (*P. patens*) were identified and obtained from the HMMER web server (http://hmmer.janelia.org/)^55^ by using the *SrMFT* gene sequence as the query and selecting sequences with the lowest E values. A phylogenetic tree of the full-length sequences of these proteins was constructed based on the alignment from CLUSTAL W using the maximum likelihood (ML) method in MEGA 6.06^56^. The consensus phylogenic trees are shown with bootstrap values from 1000 replications.

### Semi-quantitative RT-PCR of *SrFT, SrMFT*, and *SrTFL2* transcripts

Total RNA was extracted from the spadices, leaves, roots, and spathes of *S. renifolius* using an RNeasy Plant Mini Kit (QIAGEN), and cDNA was synthesized from 1 μg total RNA using a PrimeScript^TM^ RT reagent kit (Takara Bio Inc.). the resulting cDNA was then used as the template in subsequent RT-PCR experiments using ExTaq DNA polymerase (Takara Bio Inc.) with primers SrFT-F4 and SrFT-R4 for amplifying a 600-bp region containing the *SrFT gene*, SrMFT-F3 and SrMFT-R3 for amplifying a 177-bp region within *SrMFT*, and SrTFL2-F3 and SrTFL2-R3 for amplifying a 152-bp region within *SrTFL2*. As a control, the primers qPCR60-F and qPCR60-R were used to amplify a 160-bp region containing the *SrEF1α* gene. The primers SrFT-F3 and SrFT-R3 were used to amplify a 190-bp region within *SrFT* when examining the mRNA levels of cultured *S. renifolius* leaves. The primers SrFT-F1-rt and SrFT-R1-rt were used to amplify a 142-bp region within *SrFT* when examining the mRNA levels of Arabidopsis plants transformed with *SrFT*.

### *In situ* hybridization of *SrFT* transcripts

To generate an antisense probe construct, 600-bp target regions containing the *SrFT* coding sequence (525 bp) and the 5′ and 3′ flanking sequences (21 bp and 54 bp, respectively) were amplified from *S. renifolius* leaf cDNA using KOD-Plus DNA polymerase (TOYOBO) and the primers revSrFT-f1 and revSrFT-r1. The antisense probe construct was then used as a template to generate a sense probe construct, and 600-bp regions were amplified using the orSrFT-f1 and orSrFT-r1 primers. Amplified DNA fragments were inserted into the *Pst*I-*Xho*I site of the pBlueScript SK(+) vector and sequenced. *In situ* hybridization was subsequently conducted as previously described using a Hybrimaster HS-300 (ALOKA)^57^,^58^. DIG-labeled antisense and sense probes were then generated using a T3/T7 digoxigenin RNA labeling kit (Roche Diagnostics) and were hybridized to cross-sections of spadices and leaves from female-stage *S. renifolius*. Tissue sections were observed with a light microscope (SteREO Discovery V20; Carl Zeiss).

### SDS-PAGE and immunoblotting

SDS-PAGE was carried out as described previously^59^. After electrophoretic separation using SDS-PAGE, proteins were electrotransferred to a PVDF membrane (Millipore) using a semi-dry electrophoretic transfer system (ATTO) and blotting buffer (50 mM TRIS-HCl, 1.44% glycine, 20% methanol). Rabbit polyclonal antibodies against GFP were produced using His-tagged GFP as the antigen. The anti-GFP IgG was affinity-purified using a GFP antigen column and the purified IgG was used as the primary antibody to detect *SrFT-GFP* and *SrMFT-GFP*. Horseradish peroxidase-conjugated secondary antibodies were detected using SuperSignal West Femto Maximum Sensitivity Substrate (Thermo Scientific).

### Microarray analysis

Total RNA was isolated from individual floral tissues including petals, pistils, stamens, and the pith using an RNeasy Plant Mini Kit (QIAGEN). The RNA samples were quantified using an ND-1000 spectrophotometer (NanoDrop Technologies) and the quality was confirmed with an Experion System (Bio-Rad Laboratories). The cRNA was then amplified, labeled, and hybridized to a 4 × 44k Agilent 60-mer custom oligomicroarray according to the manufacturer’s instructions. The probes on this custom array were designed based on our in-house cDNA database, which was prepared from female-stage spadices of *S. renifolius*. All hybridized microarray slides were scanned using a G2505B Microarray Scanner (Agilent). Relative hybridization intensities and background hybridization values were calculated using Agilent Feature Extraction Software (9.5.1.1), and the microarray data have been deposited in the NCBI Gene Expression Omnibus repository with the accession number GSE68011.

### Cluster analysis

To identify genes that were co-regulated with *SrFT* and *SrAOX*, cluster analysis was performed^60^, using 27,184 genes from floral tissues including petals, pistils, stamens, and the pith, and the “Correlation” clustering algorithm. According to their expression profiles, 6523, 7259, 7867, and 5535 genes were classified into four clusters I, II, III and IV, respectively.

### Gene ontology analysis

The Arabidopsis dataset for BLAST analysis was obtained from the Arabidopsis information Resource (TAIR) website (http://www.arabidopsis.org/index.jsp), and the *S. renifolius* dataset prepared in this study was analyzed using the Blast2.2.27+ program from NCBI. A homolog search was performed by using tBLASTx to search the Arabidopsis dataset for sequences that were similar to those in the *S. renifolius* dataset (DB: *Arabidopsis thaliana*, query: *S. renifolius*), and *vice versa* (DB: *S. renifolius*, query: *A. thaliana*). The genes that were among the top hits of both homolog searches and that had E values less than e^−10^ were selected, and AGI codes for the selected Arabidopsis orthologs were obtained from the TAIR database. Of 27,184 genes, a total of 8279 were annotated with AGI codes and analyzed using the Gene Ontology tool on the TAIR website, and 2052, 1981, 2438, and 1808 of the genes were classified in clusters I, II, III, and IV, respectively.

## Additional Information

**How to cite this article**: Ito-Inaba, Y. *et al*. Characterization of two *PEBP* genes, *SrFT* and *SrMFT*, in thermogenic skunk cabbage (*Symplocarpus renifolius*). *Sci. Rep.* **6**, 29440; doi: 10.1038/srep29440 (2016).

## Supplementary Material

Supplementary Information

Supplementary Information

## Figures and Tables

**Table 1:** Measurements of flowering time in transgenic *Arabidopsis* plants overexpressing *SrFT, SrFT-GFP, SrMFT*, and *SrMFT-GFP*.

Genotype	Nos of plants^a^	Days to bolting^b^	Numbers of rosette leaves^c^
*WT 1-6*	10	42.40 ± 1.08	23.10 ± 2.77
*SrFT 4-1*	10	24.30 ± 0.82	3.60 ± 0.52
*SrFT 10-3*	10	24.40 ± 0.52	3.90 ± 0.32
*SrFT-GFP 12-3*	10	24.00 ± 0.94	3.90 ± 0.57
*SrFT-GFP 14-1*	10	31.70 ± 1.83	9.50 ± 2.32
*SrMFT 6-8*	10	45.10 ± 2.69	23.5 ± 2.87
*SrMFT 14-3*	10	40.50 ± 1.58	20.2 ± 2.78
*SrMFT-GFP 4-6*	10	40.30 ± 2.60	17.3 ± 1.41
*SrMFT-GFP 16-10*	10	43.30 ± 2.54	21.4 ± 2.55

^a^Plants were grown on the potted soil at 22 °C under the day length conditions at 16 h of light/8 h of darkness (long-day conditions). Data are means ± standard deviation (SD; N = 10).

^b^The number of days required for the main shoot to attain a length of 1 cm.

^c^The number of rosette leaves observed on the day when plants start bolting.
