# Supplementary material for: Characterization of two PEBP genes, SrFT and SrMFT, in thermogenic skunk cabbage (Symplocarpus renifolius)
Source: Sci Rep. 2016 Jul 8;6:29440. doi: 10.1038/srep29440 (PMC4937424; doi:10.1038/srep29440)
Supplement: Supplementary Information [file srep29440-s1.pdf]

Supplementary Figures

Title:

Characterization of two *PEBP* genes, *SrFT* and *SrMFT*, in thermogenic skunk cabbage (*Symplocarpus renifolius*)

Authors:

Yasuko Ito-Inaba*, Hiromi Masuko-Suzuki, Haruhiko Maekawa, Masao Watanabe, Takehito Inaba

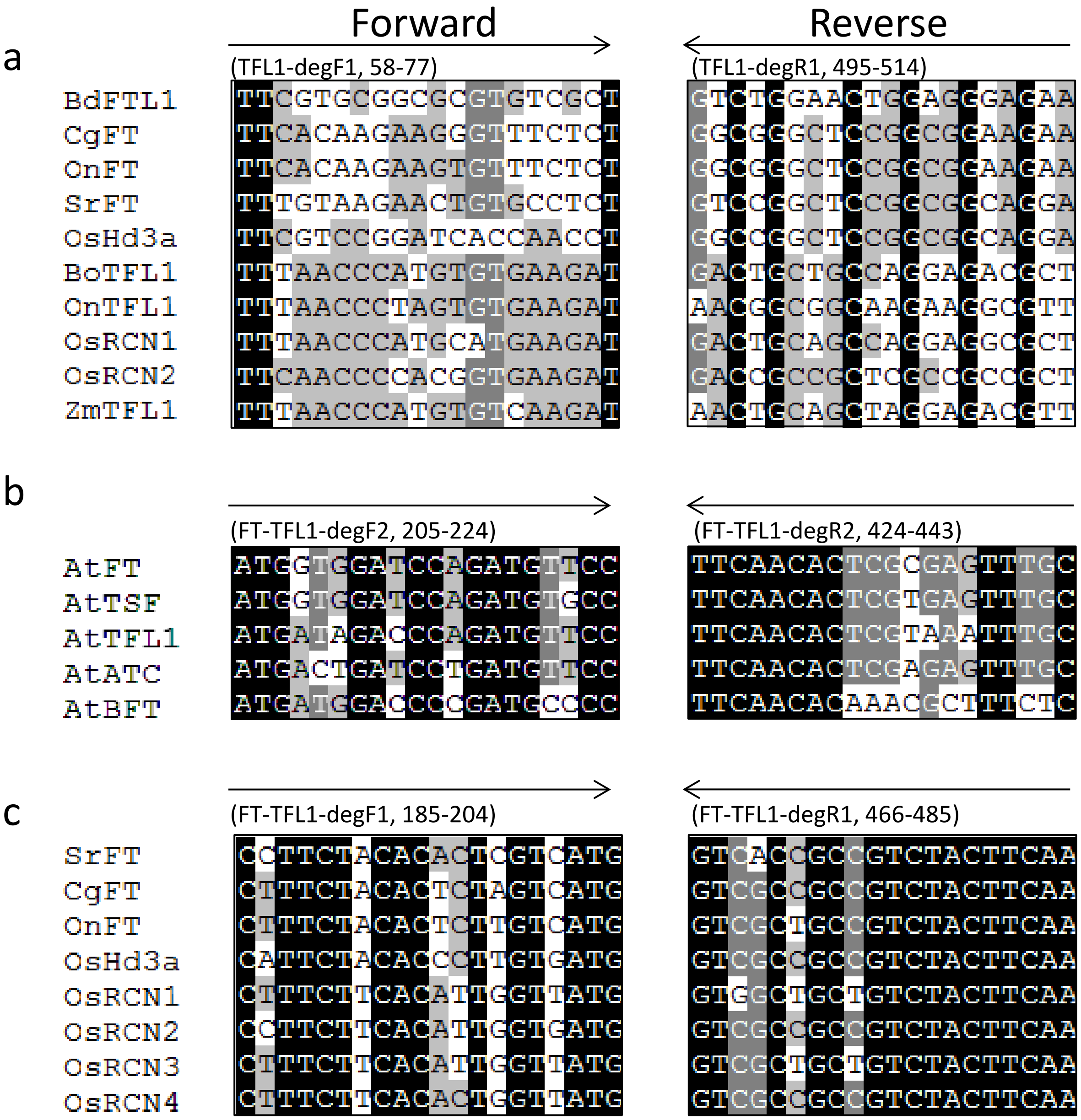

Supplementary Fig. 1. Regions used for the design of degenerate primers to isolate *TFL1*-like genes and other *PEBP* genes from *Symplocarpus renifolius*.

(a) Conserved regions specific for monocot *FT* and *TFL1* genes, respectively. Degenerate primers were designed based on the sequences of *TFL1* homologs, but not of *FT* homologs.

(b) Highly conserved regions within arabidopsis *PEBP* genes. Degenerate primers were designed based on the genes.

(c) Highly conserved regions within monocot *FT/TFL1* genes. Degenerate primers were designed based on the genes.

Forward and reverse primers were designed based on the sequences within left and right alignments, respectively. Primer names and sequence positions of the genes listed on the top are shown in brackets above each alignment. Primer sequences are shown in supplementary Table 1. White letters on a black background and on a gray background are nucleotide bases conserved across all or more than 80% of the genes in the alignment, respectively. Black letters on a grey background are nucleotide bases conserved across more than half of the genes in the alignment. The accession number and the gene abbreviations are listed in Supplementary Table 3.

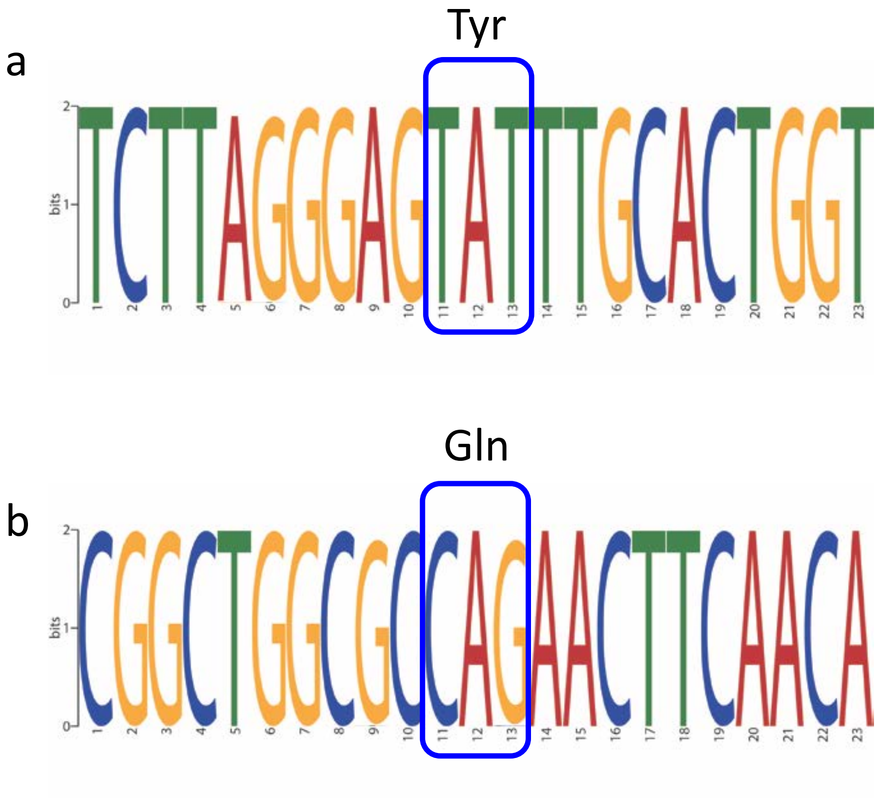

Supplementary Fig. 2. PCR amplification of the region including the key amino acids in *FT*/*TFL1* genes.
To amplify *TFL1* like genes, RT-PCR with low number of cycles (25) was performed using the degenerate primers as shown in supplementary Fig. 1c. Amplified cDNA fragments were cloned into the pZErO-2 vector, and PCR products amplified from ~100 colonies were sequenced. Tyr (a) and Gln (b) are the conserved key amino acid residues in FT homologs. In TFL1, Tyr and Gln are substituted with His and Asp, respectively. Thus, to analyze whether the clones include FT or TFL1 genes, 23 bp sequences including the 1st (a) and 2nd (b) critical codons for their key amino acid residues were analyzed. The results of the sequence alignments were displayed as simplified logos by Multiple Em for Motif Elicitaion (MEME Version 4.10.2, http://meme-suite.org/tools/meme). However, no sequences for His and Asp in TFL1 were identified, and almost all sequences were identical with *SrFT*.

```
AtTFL2   90 EGEGEGGQEERPKLDEGFYEIEAIRRKRVRKGKVQYLIKWRGWPETANTWEPLENLQSIADVIDAFEGSLKPGKPGRKRKRKYAGPHSQM 179
MdLHP1a  90 AAGGDDENRERNKLDDGFYEIEAIRRKRVRKGQLQYLIKWRGWPETANTWEPLDNLQSIADVVEAFEESLRTGKH-RKRKRKQGTPLSQP 178
SrTFL2    1 -------EGERPKLDDGYYEIEDIRKKRMRKGQLQYLIKWRGWPETANTWEPYENLHSCVDMIEAFEERLRSPKTSRKRKRKNASTHSVA 83
```

Supplementary Fig. 3. Alignment of the predicted partial amino acid sequences of SrTFL2 and related proteins.
The deduced SrTFL2 sequence was aligned with AtTFL2, *Arabidopsis thaliana* TFL2 (BAB70689) and MdLHP1a, *Malus x domestica* Borkh (AB290726). Amino acids highlighted in black and in grey are conserved across all or more than half of the protein sequences in the alignment, respectively.

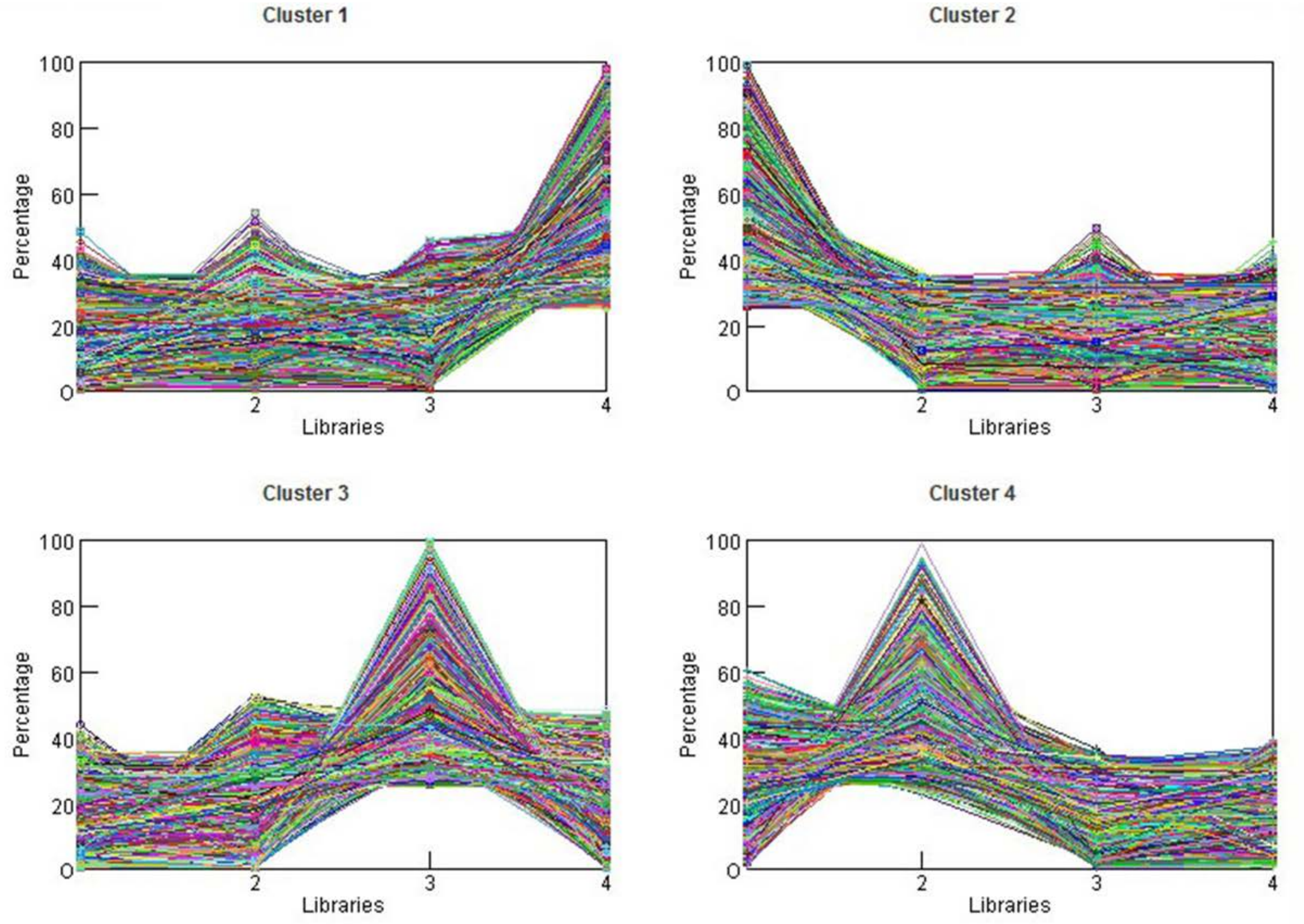

Supplementary Figure 4. Cluster analysis of floral tissue genes.
Profiles of 27,184 genes expressed within floral tissues containing petals, pistils, stamens, and pith that were organized into four clusters (I-IV). From this analysis, 6523, 7259, 7867, and 5535 genes were classified into clusters I, II, III and IV, respectively.

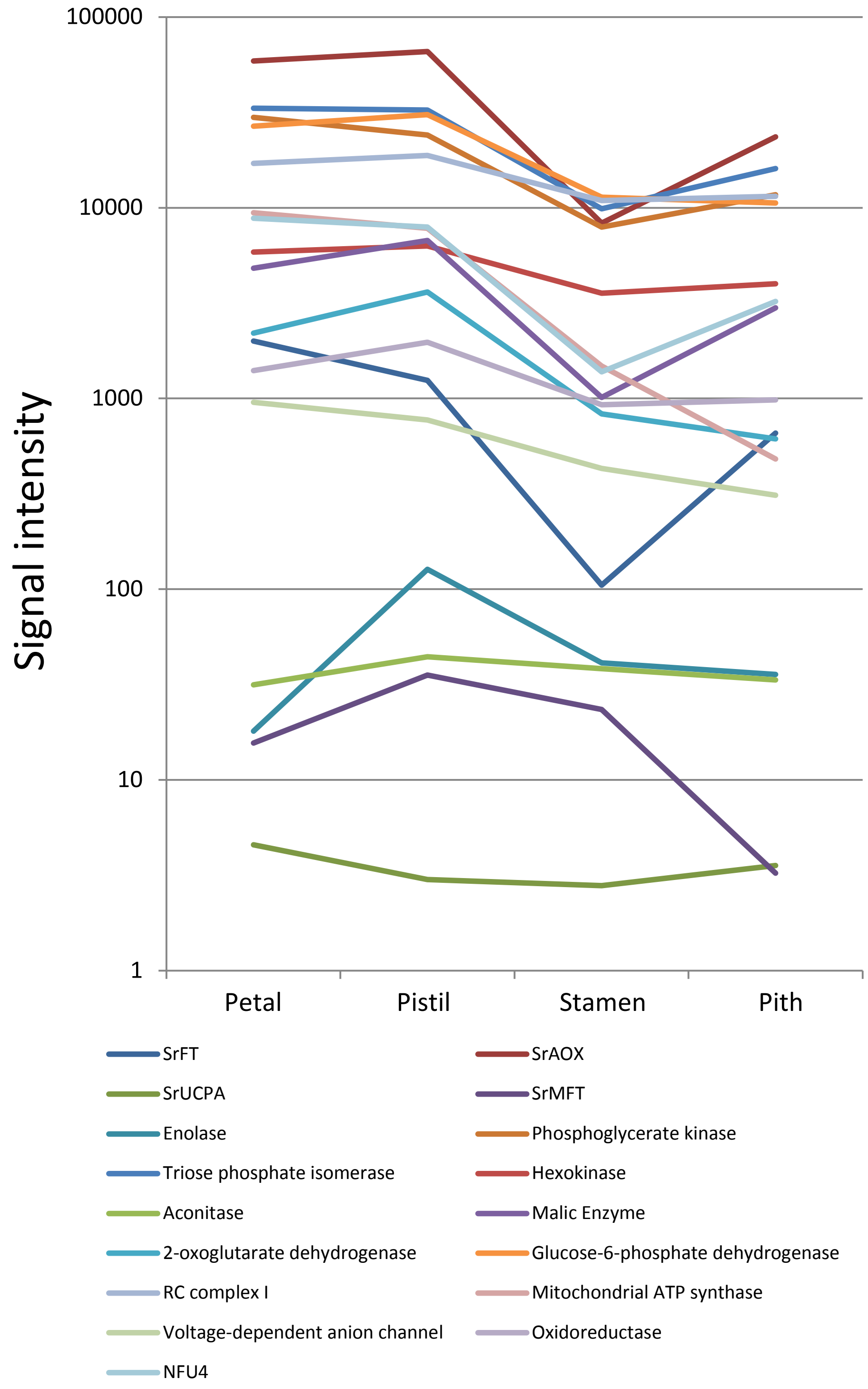

Supplementary Figure 5. Expression profiles of several genes classified into cluster IV.
Several of the genes involved in respiratory and mitochondrial function that were highly expressed in the female-stage spadices[22] were classified into cluster IV and were co-regulated with *SrFT* and *SrAOX*. *SrUCPA* was not co-regulated and was classified into cluster II.

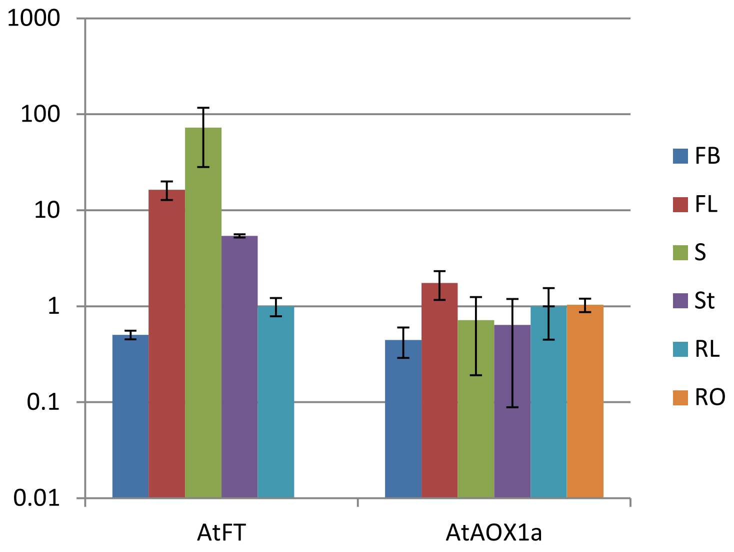

Supplementary Fig. 6. The tissue-specific expression of *AtFT* mRNA in Arabidopsis.
Quantitative RT-PCR was performed using different tissues of 52-d-old wild type Arabidopsis (ecotype Columbia) grown in soil under LD conditions (16 h light/8 h dark). FB, flower buds; FL, flowers; S, siliques; St, stems; RL, rosette leaves; Ro, roots. *FT* genes were highly expressed in flower and siliques, whereas *AOX1a* genes were expressed consistently in all organs.

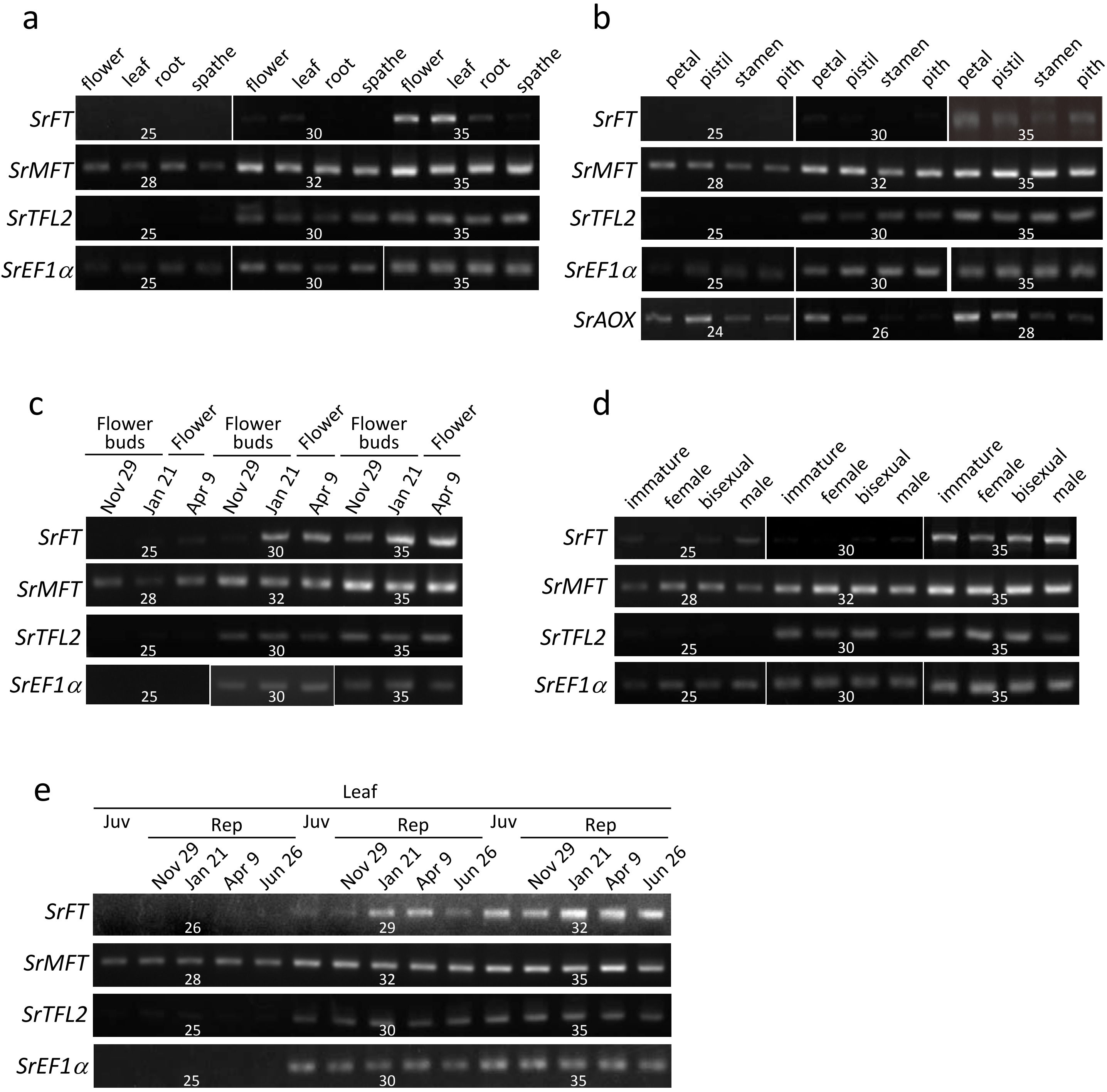

Supplementary Figure 7. Semi-quantitative RT-PCR with different PCR cycles.
Tissues or stages used here were the same as those described in Fig. 3a-e. The numbers of PCR cycles were shown in the lower part of each gel in white letters. Based on these results, semi-quantitative RT-PCR with appropriate PCR cycles were performed in Fig. 3.

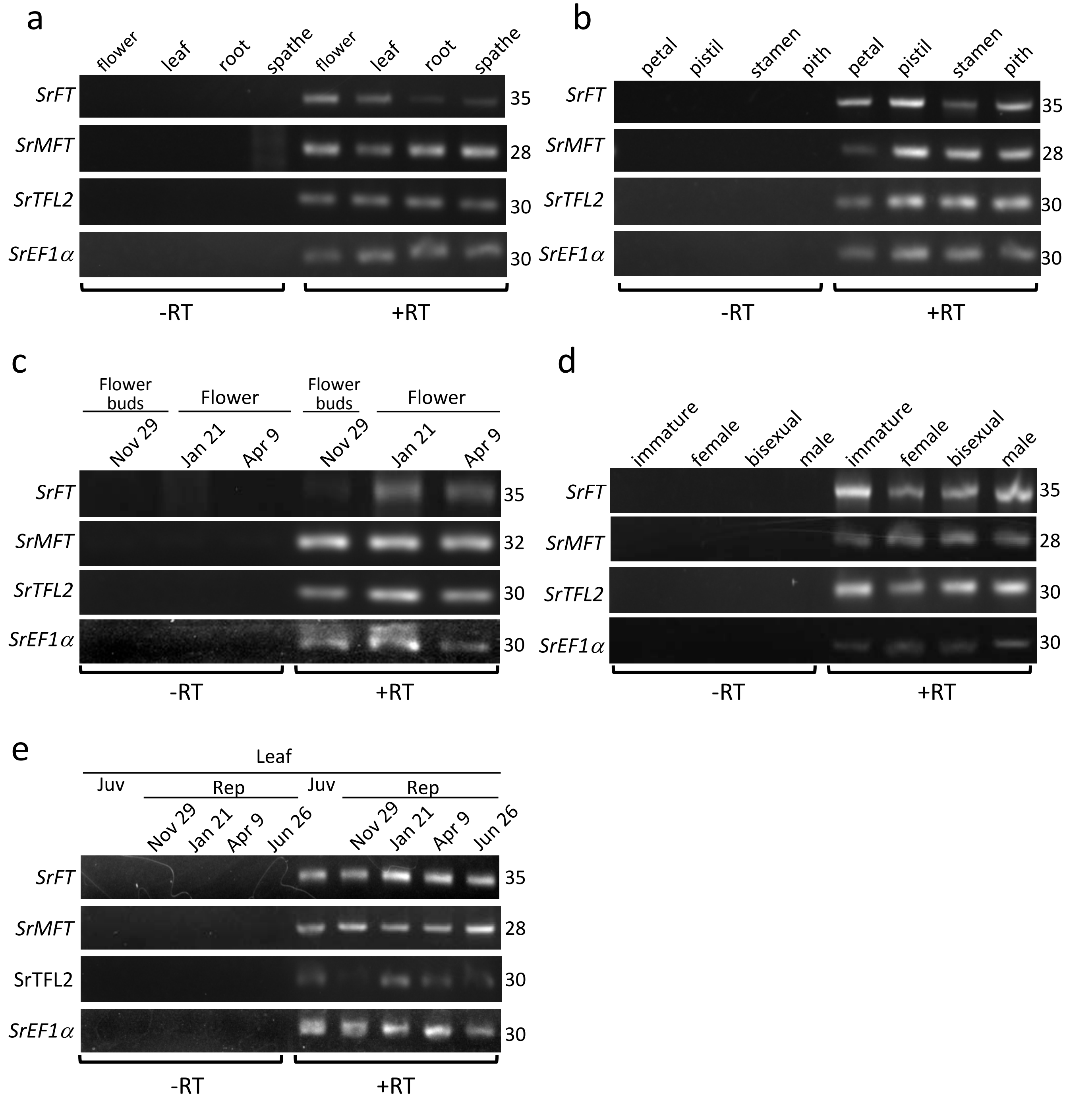

Supplementary Fig. 8. Semi-quantitative RT-PCR analyses with or without reverse transcriptase. RT-PCR analyses were performed with or without reverse transcriptase (+RT or –RT, respectively). The number of PCR cycles is shown on the right side of each gel. These data indicate that the signals detected in Fig. 3 were not from contaminated genomic DNA.

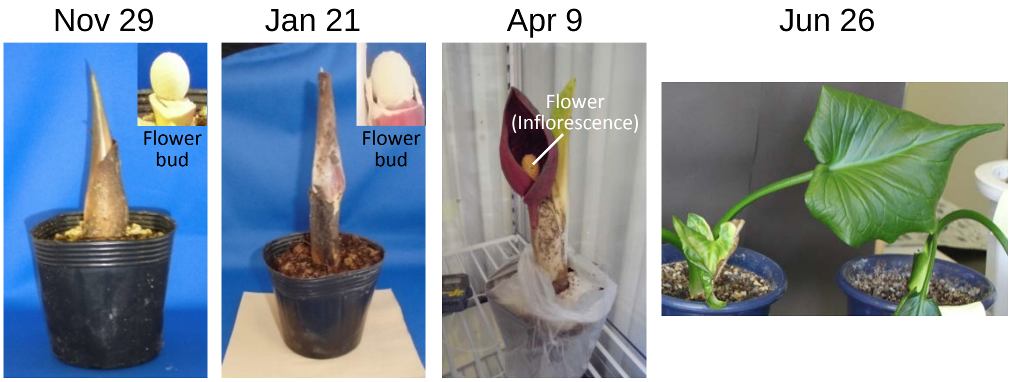

Supplementary Fig. 9. Potted plants used in the present study.
Potted plants that were transferred from outdoors at the end of autumn were used to analyze stage-specific expression of several genes during floral maturation (Fig. 3c and 3e). These potted plants allowed us to collect samples of flowers and leaves in these stages. Sampling from wild plants in these stages was impossible because a large amount of snow covered them outdoors.
